# Postoperative Stress Accelerates Atherosclerosis Through Inflammatory Remodeling of the HDL Proteome and Impaired Reverse Cholesterol Transport

**DOI:** 10.1002/advs.202511121

**Published:** 2026-01-30

**Authors:** Dominique M. Boucher, Victoria Lorant, Valerie Rochon, Abigail Carter, Cameron Stotts, My‐Anh Nguyen, Thomas Laval, Christina Emerton, Nathan Joyce, Nysa Vinayak, Marlena Scaffidi, David Cook, Katey J. Rayner, Rebecca C. Auer, Scott M. Gordon, Mireille I. Ouimet

**Affiliations:** ^1^ Department of Biochemistry Microbiology and Immunology University of Ottawa Ottawa ON Canada; ^2^ University of Ottawa Heart Institute Ottawa Ontario Canada; ^3^ Saha Cardiovascular Research Center and Department of Physiology University of Kentucky Lexington Kentucky USA; ^4^ Cancer Therapeutics Program Ottawa Hospital Research Institute Ottawa Ontario Canada; ^5^ Division of General Surgery Department of Surgery University of Ottawa Ottawa Ontario Canada

**Keywords:** acute phase response, atherosclerosis, HDL, reverse cholesterol transport, surgery

## Abstract

Over 10 million patients undergoing non‐cardiac surgery each year face major cardiovascular complications within 30 days, many due to destabilized atherosclerotic plaques. Reverse cholesterol transport (RCT), driven by HDL and Apoa1, protects against plaque progression, but the effects of surgical inflammation on this pathway remain unclear. Using an abdominal laparotomy model in *ApoE^−/−^* mice on a Western diet, we isolated the impact of surgical inflammation without confounding blood loss. Surgery acutely impaired RCT and cholesterol efflux, with inflammatory remodeling of HDL marked by elevated SAA1/2 and reduced Apoa1. Plaques exhibited higher intracellular lipids, PLIN2 expression, and cleaved caspase‐3, indicating lipid‐driven apoptosis. Both leukocytic and non‐leukocytic foam cells showed increased PLIN2, with apoptosis concentrated in PLIN2^hi^ cells. Using a novel dual‐label, dual‐cell‐type in vivo RCT model, we found that surgery significantly impaired macrophage RCT while VSMC RCT remained largely unaffected, highlighting foam cell subtype‐specific vulnerability to surgical inflammation. These findings were mirrored in general surgery patients, whose postoperative plasma exhibited markedly reduced cholesterol efflux capacity. In mice, rh‐APOA1 treatment partially restored RCT and reduced plaque lipid accumulation. Surgical inflammation rapidly disrupts HDL function and RCT, promoting foam cell apoptosis and plaque destabilization. Timely Apoa1 restoration may help reduce postoperative cardiovascular risk.

## Graphical abstract

The study shows that noncardiac surgical inflammation rapidly disrupts HDL function and cholesterol efflux in mice and human patients. Impaired reverse cholesterol transport after surgery drives rapid lipid accumulation, NETosis, and apoptosis within atherosclerotic plaques. This acute remodeling heightens plaque vulnerability by 15 days post‐surgery. Enhancing cholesterol transport with rh‐APOA1 emerges as a promising strategy to counter surgery‐induced plaque destabilization.

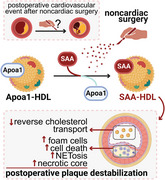

## 1 Introduction

Each year, over 10 million patients undergoing non‐cardiac surgery worldwide will suffer major cardiovascular complications within 30 days of their procedure [1, 2]. Even though atherosclerosis, affecting at least 30% of the general population, is a known risk factor for major postoperative cardiovascular complications and mortality [1, 2, 3, 4], the triggers for plaque disruption and rupture during the perioperative period remain unclear. Atherosclerosis is a chronic sterile inflammatory disease characterized by the build‐up of lipid‐rich plaques in the arteries. Initially, the infiltration and modification of low‐density lipoprotein (LDL) in the vascular wall elicit the recruitment of circulating monocytes, which differentiate into macrophages and engulf modified LDL, transforming into foam cells [5, 6, 7]. In parallel, a subset of neighboring vascular smooth muscle cells (VSMCs) undergoes lipid loading and phenotype switching to macrophage‐like foam cells [8, 9, 10, 11, 12, 13]. The recent discovery that VSMC foam cells can make up over 70% of the foam cell population in both mouse and human atherosclerotic plaques [9, 10, 11, 12, 13] challenges the previous view that macrophages are the primary source of foam cells and underscores the need to investigate VSMC foam cells in atherosclerosis.

In atherosclerosis, the balance of pro‐inflammatory and inflammation‐resolving mechanisms dictates the final clinical outcome [14]. Cholesterol efflux pathways exert antiatherogenic and anti‐inflammatory effects by reducing foam cell cholesterol accumulation and by limiting activation of the NLRP3 inflammasome [15, 16]. In parallel, efferocytosis, the clearance of apoptotic cells by macrophages, helps prevent secondary necrosis and stimulates the release of anti‐inflammatory cytokines [17]. In advanced atherosclerosis, inflammation‐resolving mechanisms become overwhelmed, and uncleared apoptotic cells undergo secondary necrosis, promoting inflammation and tissue damage. Additionally, macrophage‐VSMC crosstalk dictates plaque stability [18]. While pro‐resolving factors released by macrophages in early atherosclerosis promote VSMC proliferation to increase plaque stability [17], pro‐inflammatory mediators released by macrophages in late atherosclerosis cause VSMC de‐differentiation, extracellular matrix degradation, and fibrous cap thinning [18]. Ultimately, impaired inflammation resolution and expansion of the necrotic core lead to plaque disruption, acute thrombosis, and tissue ischemia or infarction [19].

The 1948 Framingham Heart Study was the first to report an inverse association between plasma high‐density lipoprotein cholesterol (HDL‐C) and cardiovascular disease (CVD) risk [20]. This discovery spurred numerous studies into the mechanisms by which HDL protects against CVD, leading to the identification of the reverse cholesterol transport (RCT) pathway [21]. RCT, the net flux of cholesterol from peripheral tissues, including arterial foam cells, to the bloodstream for fecal excretion, is crucial for protecting against atherosclerosis [22]. Together, the ATP‐binding cassette (ABC) transporters ABCA1 and ABCG1 mediate the bulk of cholesterol efflux to HDL, with ABCA1 transferring cholesterol to apolipoprotein A‐I (Apoa1) and small HDL particles, and ABCG1 facilitating efflux to mature HDL [23, 24, 25, 26, 27, 28]. While negative HDL‐raising clinical trials have cast doubt on the ‘HDL hypothesis’, it has become clear that plasma HDL‐C levels per se are not a faithful biomarker of CVD and do not accurately reflect HDL particle abundance, the distribution of HDL subspecies, or the ability of HDL to mediate RCT [22]. Indeed, large clinical studies showed that HDL cholesterol efflux capacity (CEC) has a stronger inverse relationship with CVD risk than HDL‐C levels, even after adjusting for HDL‐C [29, 30, 31, 32], with only one study reporting otherwise [33].

Strong evidence supports HDL's anti‐inflammatory role beyond RCT, contributing to its anti‐atherosclerotic effects [34, 35, 36]. Meta‐analysis of HDL proteomes from 45 studies identified many inflammation‐related proteins, including acute phase response (APR) proteins, serum amyloid A (SAA) 1 and 2, indicating HDL's broader functions [34]. Acute inflammation can induce changes in HDL composition and metabolism [37], impairing RCT [38, 39]. Mechanistically, acute inflammation markedly raises HDL SAA levels, displacing Apoa1 and reducing HDL CEC, while SAA deletion in mice preserves efflux capacity [38, 39, 40]. These studies linking impaired RCT and inflammation, along with the notable enrichment of APR proteins on HDL particles in CVD patients, suggest that impaired RCT due to inflammatory remodeling of the HDL proteome contributes to atherosclerosis progression.

Few studies have investigated the impact of postoperative stress on HDL, RCT, and atherosclerosis. Janssen et al. (2015) first showed in a mouse model that perioperative stress increases plaque volume and vulnerability [41]. This finding was further corroborated by Fuijkschot et al. (2016) and Handke et al. (2021), who observed increased plaque necrosis and size after surgery [42, 43]. However, these studies are confounded by the combined effects of blood loss and surgery, as hemodynamic changes by themselves are linked to major adverse cardiovascular events (MACE) [44]. This study isolates the effects of surgery‐induced inflammation by utilizing an abdominal laparotomy model that minimizes blood loss and avoids perioperative blood draws. We show that postoperative stress acutely remodels HDL and impairs RCT, leading to rapid lipid accumulation, foam cell apoptosis, and necrotic core expansion in atherosclerotic plaques. Using a novel dual‐cell‐type RCT model, we demonstrate that macrophage‐derived RCT is selectively impaired, while VSMC‐derived RCT remains largely intact. Notably, immediate rh‐APOA1 supplementation at the time of surgery partially restores RCT and mitigates plaque lipid buildup, suggesting a potential therapeutic strategy to reduce postoperative cardiovascular risk.

## 2 Methods

### 2.1 Data Availability 

The data supporting the findings of this study are included in the article and Supplemental Material. Any additional data will be made available by the corresponding author upon reasonable request.

### 2.2 Animals

All procedures were approved by the University of Ottawa Animal Care and Use Committee. Male and female *ApoE^−/−^* mice (B6.129P2‐*Apoe^1Unc^*/J; strain 002052) were acquired from Jackson Laboratory and kept on a normal laboratory diet (Envigo; 2019) in a temperature and light‐controlled environment. The animals were started on a Western diet (WD; Envigo; TD.88137, 0.2% cholesterol) at 8 weeks of age to accelerate atherosclerosis development. C57BL/6 mice were obtained from Charles River Laboratories for preliminary studies (C57BL/6N), and from Jackson Laboratories (C57BL/6J) when used as cell donors for RCT experiments in *ApoE^−/−^* animals (Figures 4 and 5).

### 2.3 Abdominal Laparotomy

8‐week‐old *ApoE^−/−^* mice were fed a WD for 8 weeks and subjected to an exploratory abdominal laparotomy. Mice received 1.2 mg/kg buprenorphine SR subcutaneously 1 h before a 30‐min surgery under 2–3% isoflurane sedation on a warming surface. A 4 cm midline incision was made, intestines were gently manipulated in four locations using a saline‐soaked cotton Q‐tip (5 strokes/quadrant), before closing with sutures. Fluid support in the form of 200 µL of subcutaneous warm 0.9% saline was provided. Anesthesia‐only control mice were similarly sedated and received 200 µL of subcutaneous warm 0.9% saline. Animals were monitored per institutional guidelines following their procedure to confirm appropriate recovery. Animals were randomized into baseline, surgery, or anesthesia control groups based on weights and plasma cholesterol (assessed using the *Infinity* cholesterol liquid stable reagent, Thermo Scientific). The baseline group was sacrificed on the day of the surgeries, whilst anesthesia control and surgery groups were sacrificed on postoperative day 1, 3, or 15 for atherosclerosis studies.

### 2.4 Plasma Cytokine Quantification

Plasma SAA from individual animals was assessed using a Mouse SAA ELISA Kit (Invitrogen), as per the manufacturer's protocol. Other inflammatory cytokines were assessed with the LEGENDplex Mouse Inflammation Panel 13‐plex (Biolegend), pooling plasma of 3–5 mice from each condition and running in technical duplicates.

### 2.5 Lipoprotein Profiling

Lipoprotein fractions were obtained through FPLC separation of 200 µL plasma using a Superose 6 Increase column at a flow rate 0.75 mL/min. Following sample injection, 500 µL fractions were collected and assessed for total cholesterol (Pointe Scientific), phospholipids (Fujifilm), and triglycerides (Fujifilm). Lipoprotein fractions were defined as follows: VLDL fractions 3–7, LDL fractions 8–15, HDL fractions 16–20. 200 µL of each HDL fraction was combined to create an HDL pool for HDL proteomics and cholesterol efflux studies.

### 2.6 Histological Analyses

All histological assessments were conducted in accordance with the American Heart Association guidelines [45]. Aortic roots were collected at sacrifice, embedded in optimal cutting temperature (OCT) medium, flash frozen, and stored at −80°C until sectioning. The aortic sinuses were serially sectioned at 10 µm thickness and spaced 100 µm apart. Sections were stored at −20°C until use. Hematoxylin and eosin (H&E), Oil Red O (ORO), and Masson's Trichrome (MT) staining were performed to evaluate plaque area, necrotic area (defined as acellular regions), neutral lipid content, and collagen deposition, respectively. Slides were imaged using a Leica Aperio 8 slide scanner with an HC (high capacity) Plan‐Apochromat 20x/0.8 objective. Image analysis was performed using the Fiji software [46].

### 2.7 Immunophenotyping

50 µL of whole blood was collected by cardiac puncture in EDTA‐coated tubes and stained immediately for flow cytometry. Red blood cells were lysed using PharmLyse (BD Biosciences). Fc block was performed for 5 min (BD Biosciences) in Brilliant Stain Buffer (BD Biosciences). An antibody mastermix was added and incubated for 20 min at 4°C, before adding Fixable Viability Stain 700 and incubating for an additional 30 min at 4°C. The cells were fixed with Cytofix (BD Biosciences) and analysed on a BD LSRFortessa or a BD FACSAria III. The immune cell populations were defined as follows (Figure S1a): leukocytes CD45^+^; inflammatory monocytes CD11b^+^ Ly6G^−^ Ly6C^hi^; neutrophils CD11b^+^ Ly6G^+^. The analysis was performed using FlowJo V10.10 (Becton Dickinson). A complete list of antibodies is available in the data supplemental (Table S1).

To define HSPC populations, bone marrow (BM) was collected by flushing femurs and tibiae with Flow Cytometry Buffer (PBS + 2% FBS). Spleens were mechanically dissociated through a 40‐µm strainer using the same buffer. Lineage depletion was performed on 1 × 10^8^ cells per sample using the EasySep Mouse Hematopoietic Progenitor Cell Isolation Kit (STEMCELL Technologies). Cells were incubated with Fc block and isolation cocktail for 15 min, followed by the addition of RapidSpheres according to the manufacturer's instructions. After magnetic separation, lineage‐depleted cells were washed and counted. 1 × 10^6^ cells per sample were pelleted and resuspended in Fc block or CD16/32‐PerCP‐Cy5.5 for 5 min at RT. Cells were stained for 20 min at 4°C for CD117 (BV605), CD34 (BV421), CD16/32 (PerCP‐Cy5.5), Sca‐1 (PE‐Cy7), CD135 (PE‐CF594), CD150 (PE), CD48 (APC). Live/dead staining was performed using Fixable Viability Stain 700 for 30 min at 4°C. Following washing, cells were fixed with Cytofix for 15 min at 4°C, washed twice in flow cytometry buffer, and stored overnight at 4°C protected from light. Samples were acquired on a BD FACSAria III cytometer.

### 2.8 Aortic Digest and Flow Cytometry Staining

Mice were perfused with HBSS, and the aortic arches and branches (brachiocephalic, left common carotid, and left subclavian arteries) were dissected and cleaned of all surrounding tissue. Aortic arches were cut into small pieces, and enzymatically digested with 0.4 units/mL Liberase (Roche), 40 units/mL hyaluronidase (Sigma), and 20 units/mL DNAse (Sigma) in HBSS (1.4 µm CaCl_2_, 1 mm EDTA) for 15–20 min at 37°C. The cell suspension was passed through a 70 µm cell strainer and stained for flow cytometry. FC block was performed for 5 min in Brilliant Stain Buffer at room temperature (RT). An antibody mastermix was added and incubated for 20 min at 4°C, before adding Fixable Viability Stain 510 and incubating for an additional 30 min at 4°C. The cells were fixed with Cytofix (BD Biosciences). BODIPY staining was performed for 30 min at RT and analysed immediately after on a BD LSRFortessa. The immune cell populations were defined as follows (Figure S2): leukocytes CD45^+^; myeloid cells CD45^+^CD11b^+^. The analysis was performed using FlowJo V10.10 (Becton Dickinson). A complete list of antibodies is available in the data supplemental.

### 2.9 Immunohistochemistry

#### 2.9.1 PLIN2 / Cleaved Caspase‐3 Panel

Frozen aortic root sections were air‐dried for 20 min at RT under air flow and fixed in 4% PFA for 10 min at RT. Slides were washed thrice for 5 min in PBS, then blocked in 10% horse serum + 0.1% Triton X‐100 at RT. Primary antibodies were prepared in 1% horse serum + 0.1% Triton X‐100, and incubated overnight at 4°C in a moisture chamber. After three PBS washes, secondary antibodies (prepared in 1% horse serum + 0.1% Triton X‐100) were added and incubated for 1 h at RT. Slides were washed three times in PBS and incubated with the primary conjugated antibody for 2 h at RT. Following three PBS washes, sections were counterstained with Hoechst 3342, mounted in ProLong^TM^ glass antifade mounting medium, and allowed to dry overnight.

#### 2.9.2 NET / Apoa1 Panel

Frozen aortic root sections were air‐dried for 20 min at RT under airflow, fixed in ice‐cold acetone for 10 min, and air‐dried again for 10 min under airflow. staining was performed as described above, with the following modifications: slides were blocked in 10% horse serum, antibodies were prepared in 1% horse serum, and primary antibodies were incubated at RT for 2 h.

#### 2.9.3 Imaging and Analyses

Images were acquired using a Zeiss Axio Observer.Z1 / 7 equipped with a Plan‐Apochromat 20x/0.8 M27 objective. Image analysis was performed using Fiji and the ZEISS V3.11 ZenDesk 2D image analysis toolkit (Figure S3a). For per‐cell quantification using the later, an average of 4,525 cells (SD = 1,746) per animal was analyzed.

### 2.10 HDL Proteomics Analysis

#### 2.10.1 Protein Digestion and Peptide Reduction

Pooled HDL fractions from 24‐h postoperative mice (*n* = 5) and anesthesia control mice (*n* = 5), isolated as described in *lipoprotein profiling*, were applied to lipid removal agent (LRA, MilliporeSigma Supelco) and washed with 25 mm ammonium bicarbonate to remove non‐lipoprotein associated proteins. LRA‐bound lipoprotein pellets were incubated overnight at 37°C in 0.05 µg/µL sequencing‐grade trypsin (Promega) dissolved in 25 mm ammonium bicarbonate. The next morning, samples were vortexed vigorously, centrifuged at 5,000 x *g*, and supernatants (peptide digests) were collected and stored at −80°C. For peptide reduction and alkylation, 20 µg of protein, as determined by protein assay, was transferred to a new tube, and the volume was adjusted to 60 µL with 50 mm ammonium bicarbonate (ABC) containing 1% sodium deoxycholate (DOC). Peptide thiols were reduced with 0.2 mm dithiothreitol (DTT) at 37°C for 30 min, then alkylated with 0.8 mm iodoacetamide (IAA) at 37°C for 30 min in the dark. The sample was acidified, and DOC was precipitated by adding 10 µL of 50% formic acid, then centrifuged at 16,000 x *g* for 5 min at RT, and the supernatant was recovered. Peptides were purified using C18 StageTips, dried under vacuum, and resuspended in 21 µL of 2% acetonitrile (ACN) and 0.05% trifluoroacetic acid (TFA). Peptide concentration was estimated using 1 µL for absorbance readings at 205 nm (Nanodrop) and adjusted to 0.2 µg/µL with 2% ACN and 0.05% TFA.

#### 2.10.2 LC‐MS Analysis

The nanoLC‐MS/MS system comprised a U3000 NanoRSLC liquid chromatography system (ThermoScientific, Dionex Softron GmbH) in line with an Orbitrap Fusion Tribrid–ETD mass spectrometer (ThermoScientific), driven by Orbitrap Fusion Tune Application 3.3.2782.34 and equipped with a Nanospray Flex ion source with a FAIMS Pro interface. An equivalent amount of 1 µg of each sample was injected in 5 µL. Peptides were trapped at 20 µL/min in loading solvent (2% ACN / 0.05% TFA) on a 300 mm i.d. x 5 mm, C18 PepMap100, 5 mm, 100 Å precolumn cartridge (Thermo Fisher Scientific) for 5 min. The pre‐column was switched in line with a PepMap100 RSLC, C18 3 mm, 100 Å, 75 µm i.d. x 50 cm length column (Thermo Fisher Scientific), and the peptides were eluted with a linear gradient from 5–40% solvent B (A: 0.1% FA, B: 80% ACN / 0.1% FA) over 90 min at 300 nL/min, for a total run time of 120 min. Spectra were acquired using Thermo XCalibur software 4.3.73.11. Lock mass internal calibration on the m/z 445.12003 siloxane ion was used. A total cycle time of 3 sec was equally split between three methods with a Compensation Voltage (CV) of either −40, −50, or −60. For each method, full‐scan mass spectra (350–1800 m/z) were acquired in the Orbitrap using an AGC target set on Standard, a maximum injection time of 50 ms, and a mass resolution of 120,000. Each MS scan was followed by the acquisition of fragmentation MS/MS spectra of the most intense ions for a total cycle time of 1.5 s (top speed mode). The selected ions were isolated using the quadrupole analyzer in a window of 1.6 m/z and fragmented by higher‐energy collision‐induced dissociation (HCD) with 35% collision energy. The resulting fragments were detected in the ion trap with a normalized AGC target of 33% and a maximum injection time of 50 ms. Dynamic exclusion of previously fragmented peptides was set for a period of 30 sec with a tolerance of 10 ppm. This protocol ensured efficient HDL proteome characterization with high specificity and sensitivity using nanoLC‐MS/MS.

#### 2.10.3 Spectral Annotation and Label‐Free Protein Quantification

Mass spectrometry data processing and relative quantification were performed using Proteome Discoverer (Thermo Scientific, version 3.1.0.638). Raw files were searched against the UniProt *Mus musculus* protein database (version 2024‐01‐24) using Sequest HT. Search parameters included “trypsin” as the digestion enzyme with a maximum of 2 missed cleavage sites, carbamidomethylation of cysteine was set as a fixed modification, methionine oxidation, and N‐terminus protein acetylation were set as variable modifications. Mass tolerances were 10 ppm and 0.5 Da for MS and MS/MS, respectively. Peptide and protein identifications were filtered at 1% False Discovery Rate (FDR). Label‐free quantification was performed based on precursor ion intensities. Only proteins with at least 2 unique peptides and detectable intensity values in 75% of samples in at least one group were considered as quantifiable proteins. Volcano plots were generated by plotting log_2_(fold change) vs ‐log_10_(p‐value). Heatmap of differentially expressed proteins identified in both groups was generated using Heatmapper [47], and includes proteins meeting the thresholds of an FDR‐adjusted *q*‐value of < 0.05 and a |Z score| > 1.96.

#### 2.10.4 Go Biological Pathway

GO Biological Pathways were extracted using the ShinyGo 0.80 tool [48]. HDL‐associated proteins meeting a statistical significance of *p* < 0.05 from surgery (upregulated after surgery) or anesthesia‐only (downregulated after surgery) groups were fed to the program with the following specifications: FDR cutoff: 0.05; pathway min size: 15 max size: 1000. Statistical significance of included proteins was chosen to capture a broader range of differentially abundant proteins, and regulated pathways of interest were subsequently evaluated with functional assays (e.g. in vitro cholesterol efflux).

### 2.11 Cell Culture

Murine bone marrow–derived macrophages (BMDMs) were generated by harvesting bone marrow from the long bones of C57BL/6N or C57BL/6J (RCT experiments only) mice and culturing for 7 days in DMEM media supplemented with 20% L929 conditioned media (made in‐house), 10% fetal bovine serum (FBS), and 1% penicillin‐streptomycin. Cells were maintained at 37°C and 5% CO_2_. Primary murine VSMCs were generated as previously described [13], and used at passage 3–5.

### 2.12 Reverse Cholesterol Transport

For in vivo RCT assays, radiolabeled‐cholesterol‐loaded cells were injected subcutaneously into mice post‐surgery, with plasma, liver, bile, and feces assessed for cholesterol movement, as previously [49]. 8‐week‐old male *ApoE* ^−/−^ mice fed a WD for 8 weeks were randomized into surgery or anesthesia‐only groups. Aggregated LDL (agLDL, 50 µg/mL) was prepared using endotoxin‐free LDL from human plasma and incubated with [^3^H]‐cholesterol (5 µCi/mL) for 1 h at 37°C. BMDMs from age‐matched C57BL/6J mice were incubated with radiolabeled agLDL for 30 h in 10% FBS‐containing DMEM media, washed with HBSS, and equilibrated overnight in 2 mg/mL fatty acid‐free BSA media.  Cells were then washed in ice‐cold HBSS, incubated with 5 mm EDTA for 20 min at 4°C, spun at 400 x *g* for 5 min, resuspended in ice‐cold DMEM, and injected subcutaneously into mice before waking from anesthesia. An equal number of cells and CPMs was injected into each animal within the experiment. After surgery, plasma (24 and 48 h), liver (48 h), bile (48 h), and feces (48 h) were collected and analyzed for radioactivity on a Perkin Elmer 4810TR Tricarb liquid scintillation counter.

RCT from VSMCs was similarly performed; VSMCs were loaded for 48 h with methyl‐β‐cyclodextrin (mβCD)‐cholesterol (35 µg/mL) and [^3^H]‐cholesterol (5 µCi/mL) in DMEM media containing 10% FBS, equilibrated overnight in 2 mg/mL fatty acid‐free BSA media, lifted in trypsin (0.05%)—EDTA (0.002%), spun at 400 x *g* for 5 min, resuspended in ice‐cold DMEM, and injected subcutaneously into mice before waking from anesthesia.

RCT was assessed as above. For dual‐label RCT, BMDMs and VSMCs were incubated with mβCD‐cholesterol (35 µg/mL), with [^3^H]‐cholesterol (VSMCs; 5 µCi/mL) or [^14^C]‐cholesterol (BMDMs; 1 µCi/mL) for 48 h at 37°C, before incubating overnight in 2 mg/mL fatty acid‐free BSA. BMDMs and VSMCs were lifted and prepared into separate injections in ice cold DMEM. The cells were injected subcutaneously sequentially during anesthesia recovery, with BMDMs in the scruff of the neck and VSMCs 3‐cm lower. CPMs and DPMs were measured from tissues as above on a Perkin Elmer 4910TR Tricarb liquid scintillation counter with dual radiotracer detection capacity.

### 2.13 Cholesterol Efflux

Cholesterol efflux experiments were performed as previously described [50]. Briefly, BMDMs or VSMCs derived from C57BL6/N animals were incubated for 48 h with ^3^H‐cholesterol (5 µCi/mL), then incubated overnight in DMEM containing 2 mg/mL of fatty acid‐free BSA media. The next morning, the media was replaced with 2 mg/mL of fatty acid‐free BSA DMEM with or without 1% mouse plasma or 5% isolated HDL (FPLC‐isolated as above; 5% equivalent to an average of 50 µg/mL HDL across all conditions). Plasma and HDL were filter sterilized using 0.22 µm Costar Spin‐X centrifuge tube filters beforehand. Cholesterol acceptors were incubated for 6 or 24 h. BSA media containing recombinant human APOA1 (rh‐APOA1; produced in‐house) (50 µg/mL) was used as a positive control. After incubation, the supernatants were collected and spun at 1000 x *g* for 5 min to remove cell debris. The cells were washed thrice with PBS and lysed in 0.5 m NaOH. Supernatants and cell lysates were read on a Perkin Elmer 4810TR Tricarb liquid scintillation counter. Cholesterol efflux is expressed as a percentage of ^3^H‐cholesterol in the supernatant/(^3^H‐cholesterol in the supernatant + ^3^H‐cholesterol in cells) x 100%. Plasma‐specific and HDL‐specific efflux were calculated by subtracting % cholesterol efflux in 2 mg/mL BSA media‐only control wells.

### 2.14 Cholesterol Efflux to Postoperative Human Plasma

Blood samples from male and female patients undergoing cancer surgery at The Ottawa Hospital were collected on the morning of their procedure (preoperative) or on the morning following their procedure (postoperative day 1) as part of an REB‐approved research protocol (OHSN REB‐2011884‐011H). The samples were collected in sodium heparin tubes and spun at 1000 x *g* for 10 min. The upper plasma layer was collected and centrifuged again at 10 000 x *g* for 15 min. Supernatants were collected and stored at −70°C. To assess cholesterol efflux, THP‐1 cells were incubated for 48 h with ^14^C‐cholesterol (1 µCi/mL) in 10% FBS RPMI media, then equilibrated overnight in RPMI containing 2 mg/mL of fatty acid‐free BSA media. The next morning, the media was replaced with 2 mg/mL fatty‐acid‐free RPMI media containing 2% human plasma, incubated for 6 h, and collected/analyzed as described in the *Cholesterol Efflux* methods section. Plasma samples were filter sterilized beforehand using 0.22 µm Costar Spin‐X centrifuge tube filters.

### 2.15 Western Blotting

4X Laemmli Sample Buffer (Bio‐Rad) containing β‐mercaptoethanol was added to 2 µL plasma and boiled at 95°C for 5 min. Samples were run on 8–16% Criterion TGX Stain Free Pre‐cast Gels (Bio‐Rad). Proteins were transferred onto 0.45 µm PVDF membranes using the Trans‐Blot Turbo Transfer System (Bio‐Rad). Immunoblotting was performed with primary antibodies at RT for 2 h, followed by horseradish peroxidase‐conjugated secondary antibodies for 1 h at RT. Proteins were developed using Clarity (Bio‐Rad) Substrates and imaged on a ChemiDoc MP system (Bio‐Rad).

### 2.16 rh‐APOA1 Intervention

rh‐APOA1 was synthesized and purified as previously described [51]. For studies intervening with rh‐APOA1, 40 mg/kg rh‐APOA1 diluted in saline was injected intraperitoneally (IP) in a 200 µL final volume at 0 h (upon anesthesia recovery) and 24 h postoperatively. For Figure 5l‐n, rh‐APOA1 was fluorescently labelled with AF647 using Alexa Fluor 647 C2 Maleimide, following the manufacturer's instructions at a 1:1 molar ratio (Invitrogen A20347).

### 2.17 Statistical Analyses

Data are represented as mean ± SEM. Males and females were analyzed jointly, as trends were consistent between sexes. Statistical tests were performed using GraphPad Prism V10.3.1 software (GraphPad Software, Inc) using an unpaired 2‐tailed t‐test (Figures 3, 4, 5n, and Figure S4), or 2‐way ANOVA with Holm–Šídák multiple comparisons test (all others). Outliers were identified and removed using the ROUT (Robust Regression and Outlier Removal) method (Q = 1%). Outliers removed from each experiment were as follows: Figures 2, 3, 4, 5. Additionally, two samples were excluded from the Figures. 4, 5 due to insufficient material for analysis.

## 3 Results

### 3.1 Rapid Necrotic Core Expansion in Atherosclerotic Plaques Following Surgery

**FIGURE 1:**
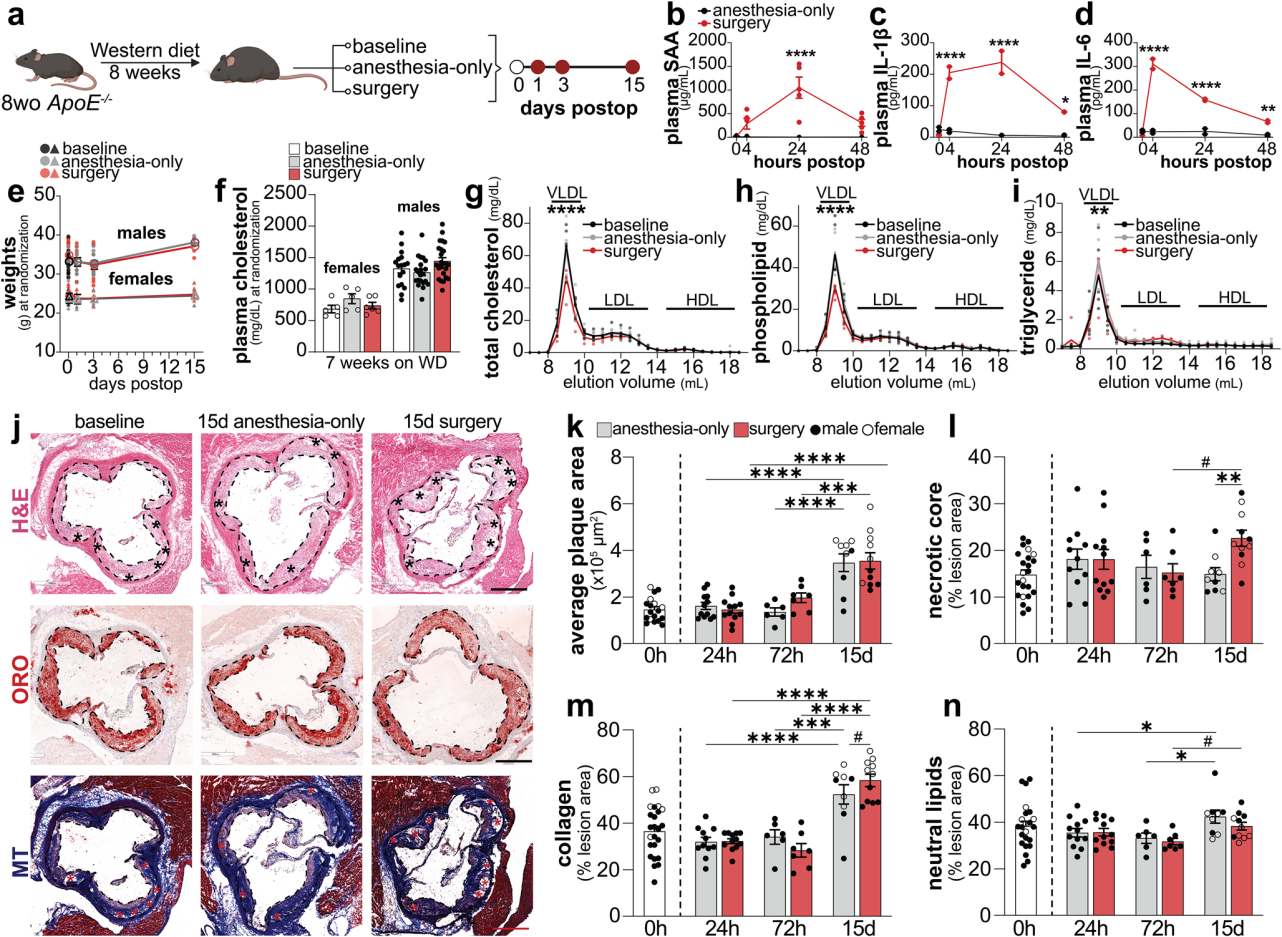
Surgery drives necrotic core expansion. 8‐week‐old *ApoE^−/−^* mice (males and females) were fed a Western diet for 8 weeks and randomised into baseline (*n* = 12), anesthesia control (*n* = 6‐12/timepoint), or surgery groups (*n* = 6‐11/timepoint) (a). (b‐d) 12‐week‐old male C57BL/6 mice underwent abdominal laparotomy (*n* = 5) or anesthesia (*n* = 3). Levels of plasma SAA (b), IL‐1β (c), and IL‐6 (d) at baseline, 4, 24, & 48 h were quantified. Mouse weights (e) and plasma cholesterol at randomization (f). Total cholesterol (g), phospholipids (h), and triglycerides (i) of HPLC‐fractionated plasma from 24 h postoperative and controls (*n* = 6 males/group). H&E‐, Oil Red O (ORO)‐, and Masson's Trichrome (MT)‐stained atherosclerotic plaques in the aortic root (necrotic regions labelled with stars) (j). Average plaque area (k), necrotic core (l), collagen (m), and neutral lipid (n), with l‐n as percentage of plaque area. Data presented as mean ± SEM. # *P* ≤ 0.1, ^*^ *P* ≤ 0.05, ^**^ *P* ≤ 0.01, ^***^ *P* ≤ 0.001, ^****^ *P* ≤ 0.0001.

Observational studies report a spike in cardiovascular events after major surgery, with increased age, male sex, and atherosclerosis as independent risk factors. However, the mechanisms of postoperative atherosclerotic plaque destabilization remain unclear. Systemic inflammation from surgery rapidly accelerates atherosclerosis in mice [41, 42, 43], but these findings are confounded by the combined effects of blood loss and surgery, as hemodynamic changes alone are linked to MACE [44]. To isolate the impact of surgery‐induced inflammation, we developed an abdominal laparotomy model with minimal blood loss and no perioperative blood draws, preserving blood volume and excluding blood loss as a confounder. In C57BL/6 male mice subjected to abdominal laparotomy, our surgical model induced an APR, with serum SAA increasing 1000‐fold, IL‐1β 200‐fold, and IL‐6 300‐fold post‐surgery, progressively returning to baseline at 48 h (Figure 1a–d).

To determine the impact of surgery‐induced inflammation on atherosclerosis using this surgery model, 8‐week‐old male and female *ApoE^−/−^* mice were placed on WD for 8 weeks and randomized into baseline, 24 h, 72 h, and 15‐day post‐surgery groups (Figure 1a,e,f). Lipoprotein profiles at 24 h post‐surgery revealed a decrease in VLDL cholesterol and phospholipids, likely related to reduced oral intake in the immediate postoperative period (Figure 1g–i). No significant differences were observed in the lipid composition of LDL or HDL, although small changes in HDL lipid composition were likely masked by low baseline levels in *ApoE^−/−^* mice (Figure 1g–i). Atherosclerotic plaques were quantified for size at the aortic root (Figure 1j). While surgery alone did not alter lesion area in the aortic sinus (Figure 1k), the necrotic core area was significantly expanded 15 days after surgery (22.6 ± 1.74% vs 14.9 ± 1.41%) (Figure 1l). Collagen deposition did not differ between the surgery and anesthesia‐only control groups (Figure 1m). Intriguingly, neutral lipids showed a downward trend, evidenced by reduced Oil Red O (ORO) staining (38.3 ± 1.67% vs 42.4 ± 2.80%) at 15 days postoperatively (Figure 1n).

### 3.2 Acute Lipid Accumulation in Arterial CD45^+^ and CD45^−^ Cells after Surgery

**FIGURE 2:**
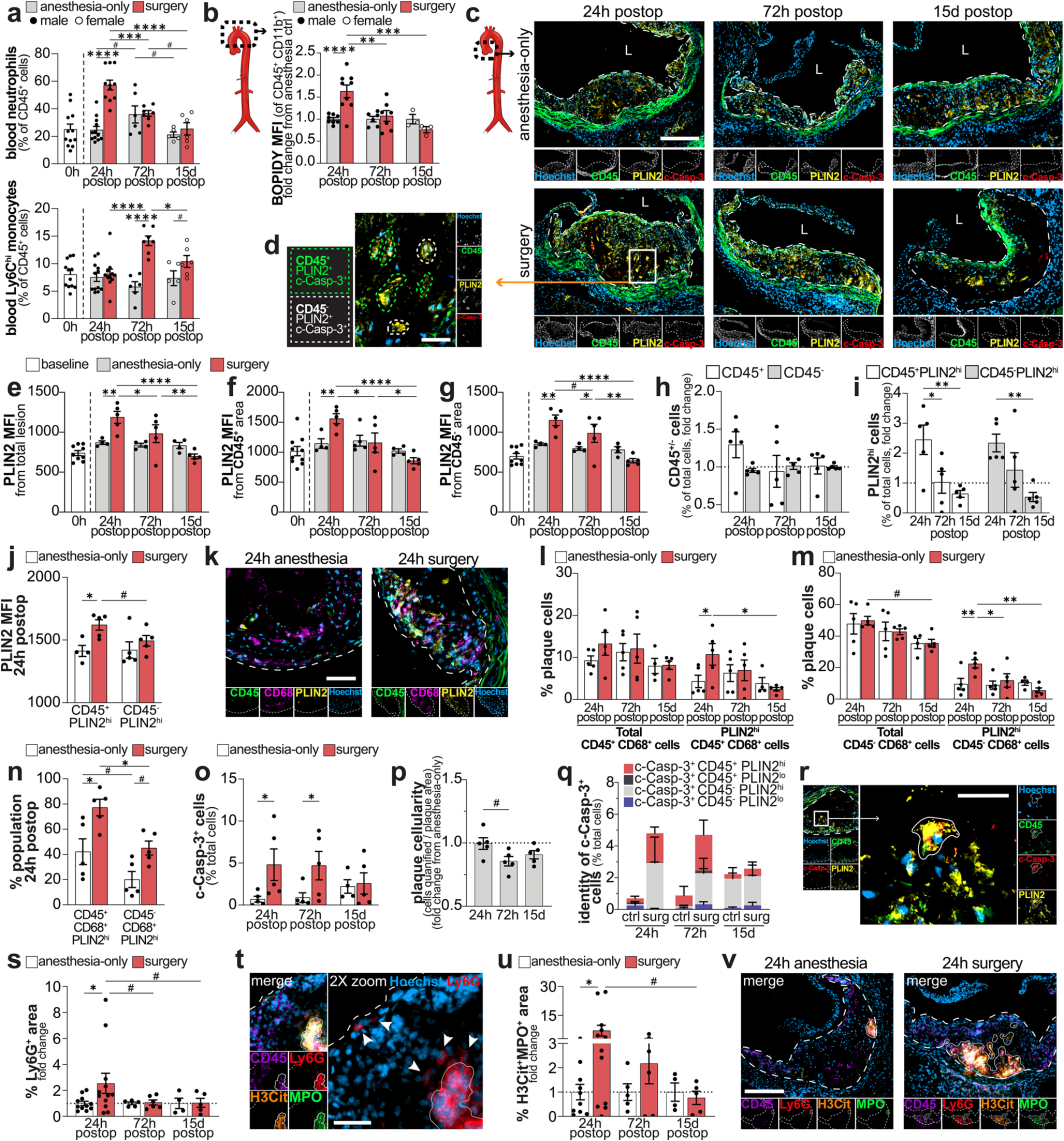
Surgery increases lipid buildup in foam cells of atherosclerotic plaques. Circulating neutrophils (CD45^+^CD11b^+^Ly6G^+^) and inflammatory monocytes (CD45^+^CD11b^+^Ly6G^−^Ly6C^hi^) in anesthesia‐only and postoperative mice (flow cytometry; *n* = 5–12/group/timepoint) (a). BODIPY MFI in aortic myeloid cells (CD45^+^CD11b^+^), as fold change from anesthesia control (b). Immunofluorescence of Hoechst, CD45, PLIN2, and c‐Casp‐3 in the aortic root (*n* = 5 males/group; scale bar: 200 µm) (c). Representative CD45^+^ (green outline) and CD45^−^ (white outline) foamy apoptotic cells (scale bar: 25 µm) (d). PLIN2 MFI in total (e), CD45^+^ (f), and CD45^−^ (g) plaque areas. Single‐cell IF analysis (h–q): CD45^+^ and CD45^−^ plaque cells (h), and CD45^+^PLIN2^hi^ and CD45^−^PLIN2^hi^ cells, as fold change from anesthesia only (i); PLIN2 MFI of PLIN2^hi^ CD45^+^ and CD45^−^ cells 24 h after surgery (j). Aortic root immunofluorescence of Hoechst, CD45, CD68, and PLIN2 (*n* = 5 males/group; scale bar: 50 µm) (k). Total and PLIN2^hi^ CD45^+^CD68^+^ macrophages (l) and CD45^−^CD68^+^ VSMCs (m), as a percentage of plaque cells. PLIN2^hi^ cells within CD45^+^CD68^+^ and CD45^−^CD68^+^ subsets 24 h post‐surgery (n). c‐Casp‐3^+^ plaque cells (o). Plaque cellularity (number of cells/plaque area), as change from anesthesia only (p). CD45 and PLIN2 expression among c‐Casp‐3^+^ cells, as percentage of plaque cells (q). Representative c‐Casp‐3^+^CD45^+^PLIN2^hi^ foam cell (scale bar: 25 µm) (r). Ly6G^+^ plaque area as fold change from anesthesia only (s). Ly6G^+^ cells around (white arrows) and within (solid outline) NET regions (scale bar: 50 µm) (t). H3Cit^+^MPO^+^ NET area (fold change from anesthesia) (u). Aortic root immunofluorescence for H3Cit, MPO, Ly6G, CD45, Hoechst; NETs outlined with solid line. Scale bar: 200 µm (v). Data are mean ± SEM. #*P* ≤ 0.1, ^*^ *P* ≤ 0.05, ^**^ *P* ≤ 0.01, ^***^ *P* ≤ 0.001, ^****^ *P* ≤ 0.0001.

Plaque destabilization due to necrotic core expansion can result from various mechanisms, including immune cell infiltration, inflammation, and lipid deposition. As expected in the postoperative period, we found increased circulating neutrophils and Ly6C^hi^ inflammatory monocytes (Figure 2a), two pro‐atherogenic cell populations. To determine whether circulating inflammatory immune cells infiltrated plaques, increasing inflammation and cellularity, and to evaluate lipid loading and foam cell formation in situ, atherosclerotic plaques were assessed in two disease‐prone regions of the aorta: the arch and the aortic sinus. In the arch, a 1.64‐fold increase in neutral lipids (per BODIPY staining) was found in the aortic myeloid cells of 24 h post‐surgery mice as compared to controls (Figure 2b).

Perilipin 2 (PLIN2), a protein found on the surface of lipid droplets that increases in expression as foam cells accumulate lipids [52], also increased in plaques of postoperative mice (Figure 2c–e). Notably, PLIN2 expression was elevated in both the CD45^+^ (Figure 2f) and CD45^−^ (Figure 2g) plaque areas of the aortic sinus 24 h after surgery. Using the ZEISS Zendesk 2D image analysis toolkit, we quantified the per‐cell expression of CD45, PLIN2, and cleaved caspase‐3 (c‐Casp‐3), a marker of apoptosis (Figure 2h–q and Figure S3). Foam cell subpopulation analysis showed a modest influx of CD45^+^ cells 24 h after surgery (Figure 2h), accompanied by increased PLIN2 expression in both leukocytic (CD45^+^) and non‐leukocytic (CD45^−^) foam cells. PLIN2 levels rose 2.45‐fold and 2.34‐fold, respectively (Figure 2i), with leukocyte‐derived foam cells exhibiting the highest PLIN2 expression (Figure 2j).

Further stratification of macrophage‐derived versus VSMC‐derived foam cells using CD68 (Figure 2k–m) revealed that the relative proportions of CD45^+^CD68^+^ (macrophage‐derived) and CD45^−^CD68^+^ (VSMC‐derived) cells remained largely unchanged between anesthesia control and surgery groups at 72 h and 15 days post‐surgery (Figure 2l,m). A slight trend toward increased CD45^+^CD68^+^ cells at 24 h (Figure 2l) parallels the 1.29‐fold rise in total CD45^+^ cells observed in Figure 2h. Although overall cellular proportions were stable, PLIN2 expression within these subsets increased following surgery, resulting in more lipid‐laden CD45^+^CD68^+^PLIN2^hi^ and CD45^−^CD68^+^PLIN2^hi^ foam cells (Figure 2l,m). After surgery, PLIN2^hi^ cells accounted for ∼75% of CD45^+^CD68^+^ cells, compared with ∼45% of the CD45^−^CD68^+^ cells (Figure 2n). Thus, although CD45^−^CD68^+^PLIN2^hi^ cells constitute a larger fraction of total plaque cells, CD45^+^CD68^+^ cells show a stronger propensity for lipid accumulation under surgical stress. In CD45^−^CD68^+^ cells, the increased lipid burden most likely reflects enhanced accumulation within existing transdifferentiated VSMCs rather than recruitment of new cells, as total cell numbers did not differ from anesthesia controls at 24 h post‐surgery (Figure 2m).

Apoptotic c‐Casp‐3^+^ cells were elevated in plaques 24 and 72 h after surgery (Figure 2o), correlating with reduced plaque cellularity at 72 h and 15 days post‐surgery (Figure 2p). These apoptotic cells were of both leukocytic and non‐leukocytic origin and were almost exclusively PLIN2^hi^ (Figure 2q). By 15 days post‐surgery, we observed reduced ORO‐positive area (Figure 1n), decreased BODIPY MFI (Figure 2b), and fewer PLIN2^hi^ cells (Figure 2e‐g,i), accompanied by an enlargement of the necrotic core, features consistent with progression toward more advanced plaque pathology. These changes likely reflect foam cell death and cellular loss following the earlier phase of lipid accumulation. Taken together, our findings reveal the remarkably rapid dynamics of lipid droplets in plaque foam cells during the APR and suggest that the exacerbation of lipid buildup in macrophage and VSMC foam cells after surgery drives necrotic core expansion.

Given the acute rise in circulating neutrophils 24 h after surgery, we next examined whether neutrophil activation and the formation of neutrophil extracellular traps (NETs), web‐like chromatin structures released by activated neutrophils, might contribute to postoperative plaque destabilization. Immunofluorescence staining of the aortic sinus revealed a marked increase in Ly6G^+^ neutrophils after surgery (Figure 2s). Ly6G signal localized around and within extracellular traps, identified by co‐positivity for citrullinated histone 3 (H3Cit) and myeloperoxidase (MPO) (Figure 2t), consistent with NET formation. NET area was significantly increased 24 h post‐surgery (Figure 2u,v), indicating that NETosis is likely a contributing mechanism to postoperative plaque destabilization.

### 3.3 Postoperative Inflammatory Remodeling of HDL

**FIGURE 3:**
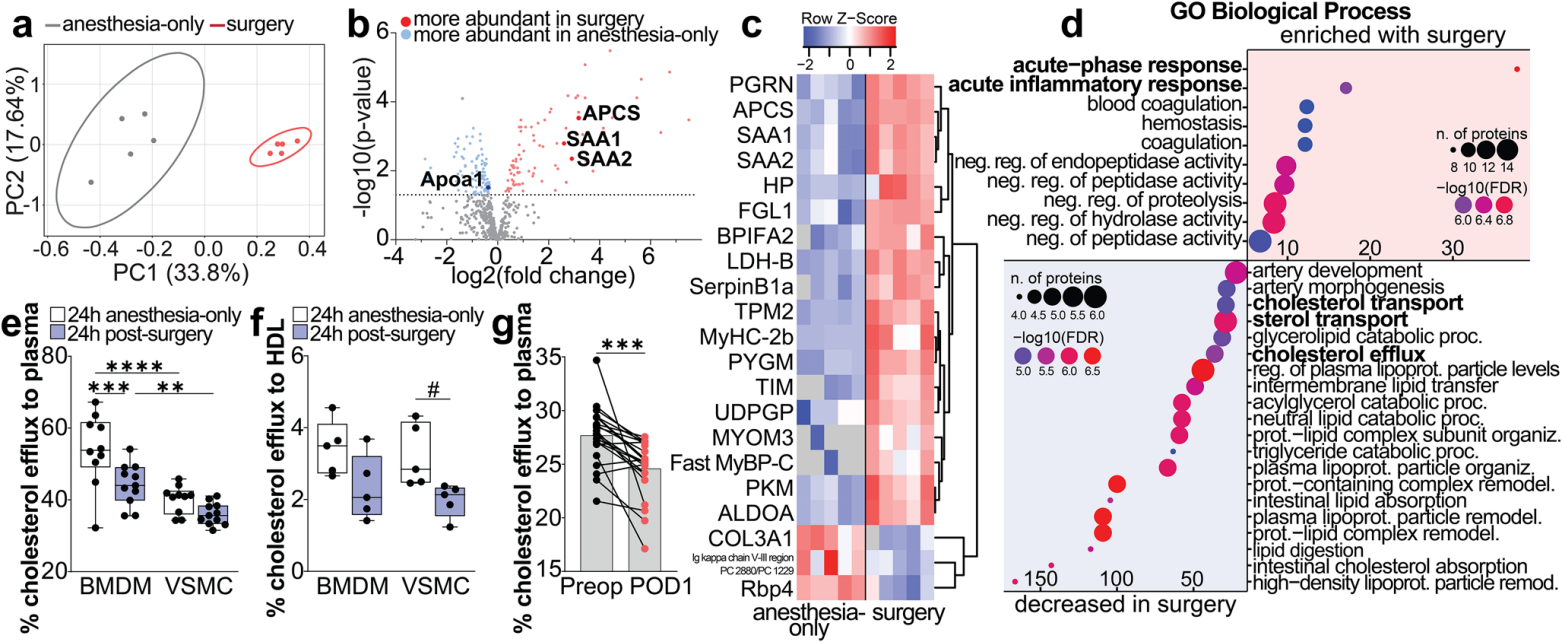
Surgery drives inflammatory remodeling of HDL, reducing cholesterol efflux capacity. HDL was isolated by HPLC from plasma of 24 h postoperative and anesthesia control mice (*n* = 5/group). The HDL proteome was analyzed by label‐free LC‐MS/MS. Principal component analysis of identified proteins (a). Volcano plot showing proteins with significantly different abundance (*p* < 0.05) (b). Heatmap of most differently abundant proteins (Limma *q*‐value < 0.05, |Z score| > 1.96) (c). GO Biological Processes of proteins significantly enriched on postoperative HDL (10 pathways presented) and enriched on anesthesia control (decreased in surgery; 20 pathways presented) (d). 24 h cholesterol efflux of BMDMs and VSMCs to 1% plasma from 24 h postoperative or anesthesia control mice (*n* = 10‐11 males/group) (e). 6 h cholesterol efflux of BMDMs and VSMCs to 5% isolated HDL (∼50 µg/mL) from 24 h postoperative or anesthesia control mice (*n* = 5 males/group) (f). ^14^C‐cholesterol efflux to preoperative (preop) or postoperative day 1 (POD1) plasma (2%) of patients undergoing general surgery (*n* = 21; lines connect results from one patient) (g). Data as min to max boxplot (e,f), # *P* ≤ 0.1, ^**^ *P* ≤ 0.01, ^***^ *P* ≤ 0.001, ^****^ *P* ≤ 0.0001 (e,f). ^***^: *P* < 0.001 (g).

Next, we used label‐free proteomics to analyze HDL proteomes of 24 h postoperative mice and anesthesia controls. Of the 392 proteins identified on HDL, 140 were differentially abundant (*p* < 0.05; 100 more abundant in surgery, 40 more abundant in control). Principal component analysis distinguished the groups (Figure 3a), with a notable increase in SAA1 and SAA2 after surgery and reduced Apoa1 (Figure 3b). Postoperative HDL was highly enriched with proteins related to the APR and acute inflammation, as indicated by significant Z‐scores (|Z| > 1.96) and GO Biological Process terms (*p* < 0.05) (Figure 3c,d). Conversely, pathways related to RCT such as cholesterol efflux and lipid transport pathways were the most downregulated in postoperative HDL (Figure 3d). Notably, cholesterol efflux from BMDMs and VSMCs to whole plasma or HPLC‐isolated HDL was significantly reduced when using samples from 24 h postoperative mice compared to those from anesthesia‐only controls (Figure 3e,f). This phenomenon was also observed in samples obtained from patients undergoing abdominal surgery for cancer, where we observed a consistent reduction in cholesterol efflux to postoperative human plasma (Figure 3g). These findings underscore a critical disruption in HDL efflux capacity following non‐cardiac surgery, highlighting a potential mechanistic link between postoperative inflammation and impaired cholesterol transport, which could have profound implications for cardiovascular health in the postoperative setting.

### 3.4 Postoperative Impairment of Reverse Cholesterol Transport

**FIGURE 4:**
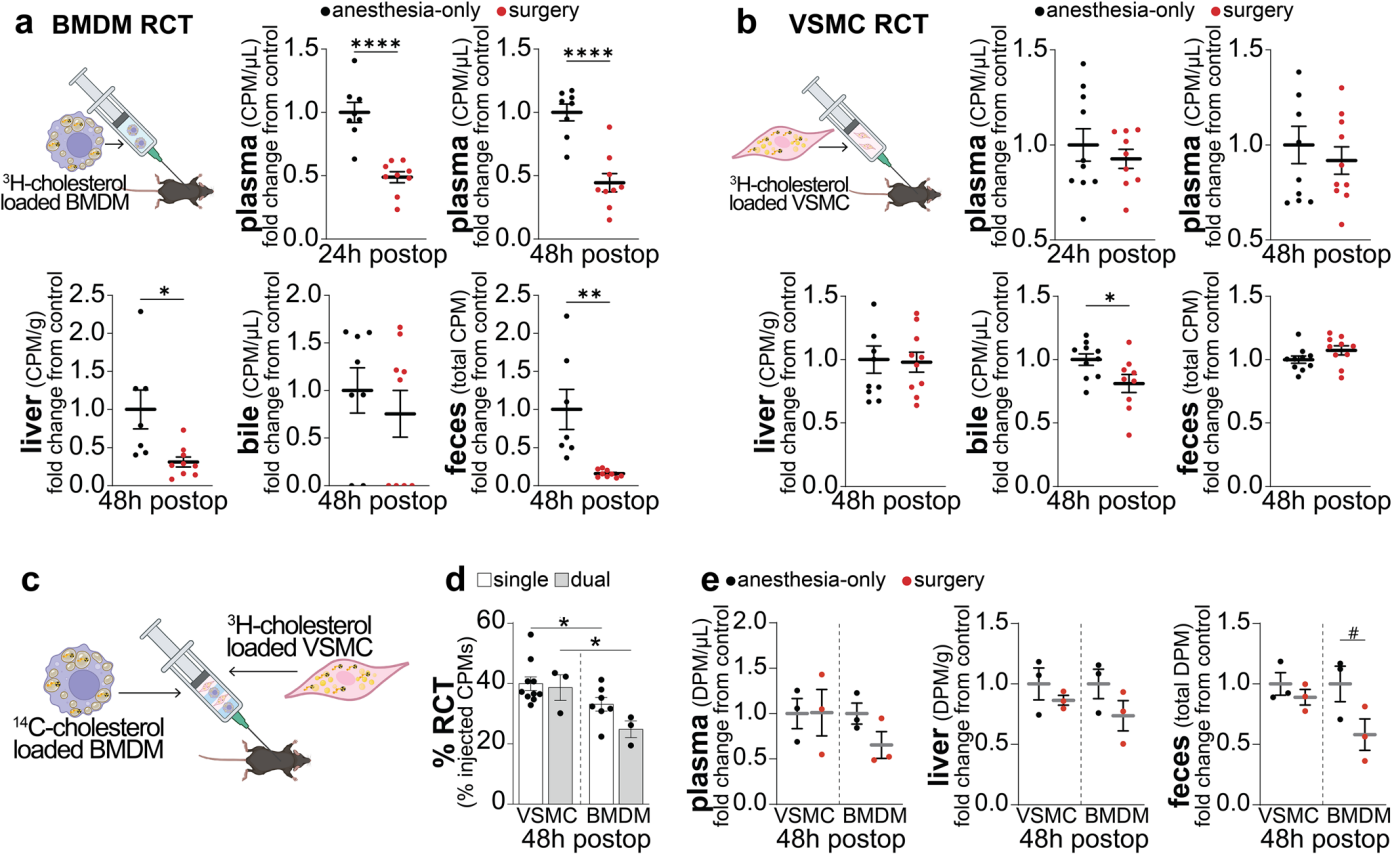
Surgery impairs macrophage RCT. Male *ApoE^−/−^* mice (*n* = 8‐9/males group) fed a Western diet for 8 weeks were randomized to anesthesia control or surgery group. BMDMs from sex‐ & aged‐matched C57BL/6 donors were loaded with ^3^H‐cholesterol‐labeled agLDL for 30 h and injected during recovery from anesthesia. Radioactivity was measured in plasma at 24 h after surgery, and in plasma, liver, bile & feces at 48 h postoperative (a). Similarly, VSMCs loaded with ^3^H‐cholesterol and mβCD‐cholesterol for 48 h were injected in mice to assess postoperative VSMC RCT (*n* = 10 males/group) (b). Experimental design for proof‐of‐concept for in vivo dual RCT study, cells were loading with mβCD‐cholesterol and radiolabeled cholesterol (^14^C for BMDMs, ^3^H for VSMCs) for 48 h before injecting postoperatively (c). Total RCT was assessed in anesthesia controls from single‐label, single‐cell studies (CPMs in quantified tissues/injected CPMs) and a dual‐label pilot study (16‐wk‐old ½ male, ½ female *ApoE^−/−^*, *n* = 3) (d). VSMC and BMDM RCT was assess simultaneously in anesthesia control and postoperative mice (16‐wk‐old ½ male, ½ female *ApoE^−/−^*, *n* = 3/group). ^3^H DPMs (VSMCs) and ^14^C DPMs (BMDMs) of 48 h plasma, liver and feces are shown (e). Data as mean ± SEM, # *P* ≤ 0.1, ^*^ *P* ≤ 0.05, ^**^ *P* ≤ 0.01, ^****^ *P* ≤ 0.0001.

To quantify RCT in vivo, 8‐week‐old male *ApoE^−/−^* mice fed a WD for 8 weeks were randomized into surgery or anesthesia‐only groups. ^3^H‐cholesterol‐loaded macrophages were injected subcutaneously before waking from anesthesia. Afterward, cholesterol movement was assessed in the plasma, liver, bile, and feces, as previously [49]. We found a marked reduction in ^3^H‐cholesterol movement from BMDMs into plasma, liver, and feces after surgery (Figure 4a). A similar impairment in macrophage RCT was observed in wild‐type C57BL/6 mice (Figure S4), indicating that postoperative inflammation suppresses macrophage cholesterol efflux and RCT independently of ApoE expression.

While macrophage RCT has been well described in mice, VSMC RCT remains largely understudied [53]. Interestingly, VSMC RCT appeared to be minimally affected by surgery, with only slight trends toward reduced cholesterol transport to plasma and bile (Figure 4b). To directly compare the RCT from both foam cell subtypes in vivo, we developed a novel dual‐label, dual‐cell‐type RCT model. Macrophages and VSMCs were incubated with mβ‐CD‐cholesterol and either ^14^C‐cholesterol (for BMDMs) or ^3^H‐cholesterol (for VSMCs) for 48 h (Figure 4c). This loading process effectively transforms VSMCs into macrophage‐like foam cells [54], allowing for equivalent lipid loading in both cell types [13]. Cells were then injected into recipient mice, and total macrophage (^14^C CPMs) and VSMC (^3^H CPMs) RCT were quantified by summing ^3^H and ^14^C CPMs across all tissues, expressing them as a percentage of total injected CPMs (Figure 4d). Results from the dual‐label, dual‐cell RCT model showed comparable RCT to the single‐label model, confirming significant VSMC RCT in vivo (Figure 4d). Once again, surgery induced significant impairment in BMDM RCT, while VSMC RCT remained largely unaffected (Figure 4e). Postoperative mice exhibited reduced BMDM versus VSMC RCT to plasma (0.65‐ vs. 1.01‐fold reduction relative to anesthesia‐only controls), liver (0.74‐ vs. 0.87‐fold), and feces (0.58‐ vs. 0.89‐fold).

### 3.5 Recombinant APOA1 Mitigates Postoperative RCT Dysfunction

**FIGURE 5:**
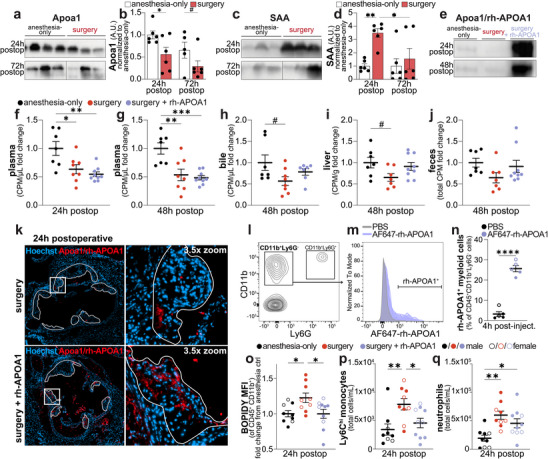
Restoring postoperative APOA1 increases RCT. Western blot of plasma Apoa1 (a,b) and SAA1+2 (c,d) (*n* = 6 males/group; 3 shown), quantified and normalized to anesthesia control (b, d). Plasma Apoa1 at 24 h & 48 h postop in mice injected IP with rh‐APOA1 (40 mg/kg) or saline at 0 and 24 h postop (e). BMDM RCT was assessed with IP rh‐APOA1 injection at 0 and 24 h postop, or equivalent volume of saline for control animals, with radioactivity measured as in Figure 4a (*n* = 7‐9 males/group) (f‐j). Aortic root immunofluorescence of APOA1 and Hoechst in 24 h post‐surgery animals injected IP with rh‐APOA1 or saline at 0 h postop (scale bar: 500 µm) (k). Myeloid (CD11b^+^Ly6G^−^) cell population in aortic arches by flow cytometry (l), myeloid cell expression of fluorescently labelled AF647‐rh‐APOA1 4 h after injection (m), and percentage of AF647‐rh‐APOA1^+^ myeloid cells (CD45^+^CD11b^+^Ly6G^−^) (n) (l‐n: *n* = 5/group, ½ males ½ females). Aortas from 24 h postop mice ± IP rh‐APOA1 at 0 h postop were digested, stained for CD45, CD11b, and BODIPY, with BODIPY MFI of CD45^+^CD11b^+^ cells normalized to anesthesia‐only controls (*n* = 5‐6 males/group) (o). Circulating inflammatory monocytes (CD45^+^CD11b^+^Ly6G^−^CD115^+^Ly6C^hi^) (p) and neutrophils (CD45^+^CD11b^+^Ly6G^+^) (q) 24 h after surgery ± IP rh‐APOA1, as absolute number (cells/mL). Data as mean ± SEM, # *P* ≤ 0.1, ^*^ *P* ≤ 0.05, ^**^ *P* ≤ 0.01, ^***^ *P* ≤ 0.001.

Following surgery, we observed marked reductions in plasma Apoa1 levels concomitant with increased SAA levels in *ApoE^−/−^* mice (Figure 5a‐d). To test if restoring Apoa1 could rescue postoperative RCT impairment, we conducted an interventional study in which mice were randomized into three groups: anesthesia‐only control + IP saline, surgery control + IP saline, or surgery + IP rh‐APOA1 (40 mg/kg in saline). IP injections were given upon waking from anesthesia and at 24 h post‐surgery. rh‐APOA1 treatment significantly increased plasma APOA1 concentrations at 24 and 48 h post‐surgery, surpassing levels observed in anesthesia‐only control levels (Figure 5e). Raising APOA1 post‐surgery modestly increased radioactive counts in the bile, liver, and feces (Figure 5f–j), indicating partial restoration of RCT.

rh‐APOA1 treatment visibly increased APOA1 abundance within aortic sinus plaques (Figure 5k). To assess whether rh‐APOA1 effectively targeted myeloid cells post‐surgery, we fluorescently labeled rh‐APOA1 with Alexa Fluor 647 and assessed its distribution in aortic arch cells 4 h after injection. More than 25% of myeloid cells were associated with AF647–rh‐APOA1 at this time point, indicating rapid uptake into the circulation and efficient delivery to atherosclerotic plaques. Notably, rh‐APOA1 reduced lipid accumulation in aortic arch myeloid cells (Figure 5l–n).

To further examine the hematopoietic response to surgical stress, we quantified hematopoietic stem and progenitor cell (HSPC) subsets and mature immune populations in the bone marrow and spleen 24 h after surgery, with or without rh‐APOA1 treatment. Surgery induced broad reductions across multiple HSPC populations (Figure S5), consistent with stress‐driven mobilization and differentiation, and these shifts were accompanied by elevated circulating mature myeloid cells (Figures 2a,b, 5p,q). Notably, rh‐APOA1 mitigated these changes, preserving several HSPC subsets in both bone marrow and spleen (Figure S5). This preservation corresponded with reduced circulating neutrophils and monocytes (Figure 5p,q), indicating that rh‐APOA1 attenuates excessive HSPC activation and dampens the surge in myeloid output induced by acute postoperative stress. Collectively, these data demonstrate that inflammatory remodeling of HDL contributes to postoperative RCT dysfunction, while raising APOA1 levels partially restores cholesterol transport and restrains stress‐induced hematopoietic activation—mechanisms that may ultimately limit lipotoxicity‐driven necrotic core expansion following surgery.

## 4 Discussion

The clinical outcome of atherosclerosis hinges on the balance between pro‐inflammatory and inflammation‐resolving mechanisms [14]. While lipid accumulation and inflammation drive plaque progression and MACE, inflammation‐resolving mechanisms help stabilize plaques, as shown by reduced MACE in trials involving IL‐1β antagonism and colchicine [7, 55, 56, 57]. HDL contributes to cardiovascular health through its anti‐inflammatory effects, including cholesterol removal from foam cells and inhibiting pro‐inflammatory pathways and cytokines such as TNFα and IL‐6 [36, 58]. While research traditionally focuses on macrophage efflux mechanisms in atherosclerosis, recent studies underscore the importance of studying VSMC‐derived foam cells, which constitute the majority of foam cells in plaques [9, 59, 60]. Therefore, understanding cholesterol efflux in VSMC‐derived foam cells is as crucial as in macrophage foam cells.

In this study, postoperative stress increased lipid accumulation in both macrophage‐ and VSMC‐derived foam cells. Although cholesterol efflux to isolated HDL was similarly reduced in both cell types, the greater reduction in macrophage efflux to postoperative plasma resulted in a significant impairment in macrophage‐derived RCT, whereas VSMC‐derived RCT showed only modest changes. One explanation is that the low baseline HDL levels in *ApoE^−^^/^^−^* mice may mask postoperative differences in VSMC RCT. In contrast, macrophages rely more heavily on ABCA1‐mediated efflux to lipid‐poor Apoa1 (pre‐β HDL) [13], making them particularly vulnerable to the marked postoperative decline in circulating Apoa1. Accordingly, efflux to total plasma decreased more in BMDMs (↓9.70%) than in VSMCs (↓4.08%), while efflux to isolated HDL declined only modestly and to a similar degree (↓1.11% in BMDMs; ↓1.25% in VSMCs). These findings support the idea that Apoa1 depletion has a greater impact on overall RCT than inflammation‐associated changes in mature HDL. Consistent with this interpretation, postoperative RCT from macrophages was substantially reduced, whereas VSMC‐derived RCT was only modestly affected. Importantly, both cell types exhibited reductions in HDL‐specific efflux, indicating that inflammatory remodeling of HDL affects efflux globally. However, our data indicate that under low‐HDL conditions, macrophage‐derived RCT is more sensitive to Apoa1 loss than VSMC‐derived RCT. Importantly, models with higher baseline HDL levels or distinct inflammatory signatures may reveal a larger postoperative impact on VSMC‐derived RCT.

Acute lipid accumulation in atherosclerotic plaques was observed in foam cells of both leukocyte and non‐leukocyte origin. Leukocyte‐derived foam cells accumulated more lipids, paralleling the cell type‐specific impairments in RCT. Although VSMC RCT was not profoundly impaired in *ApoE^−/−^* mice, VSMCs remain essential therapeutic targets given their numerical dominance within plaques. Raising postoperative Apoa1 improved macrophage RCT and reduced acute lipid accumulation, though HDL‐based approaches may provide broader support for increasing RCT across foam cell subtypes.

Macrophage‐VSMC crosstalk likely further influences plaque remodeling. In macrophages, cholesterol trafficking to the endoplasmic reticulum triggered by atherogenic LDL or loss of ABCA1/ABCG1 activates the NLRP3 inflammasome, inducing IL‐1β production [61, 62]. IL‐1β signaling promotes VSMC trans‐differentiation, inflammation and apoptosis [63]. Although overall plaque cell proportions remained stable post‐surgery, the increase in lipid‐laden CD68^+^ VSMCs suggests that heightened lipid burden occurs predominantly within pre‐existing transdifferentiated VSMCs rather than through a major change in cellular composition. Nonetheless, we cannot fully exclude increased phenotypic switching that is offset by selective apoptotic turnover. Local macrophage‐VSMC signaling may amplify lipid uptake or efferocytosis in VSMCs, and dedicated mechanistic studies are needed to elucidate the full scope of these interactions during acute inflammation.

Inflammatory IL‐1β signaling from macrophages is also likely to contribute to postoperative neutrophil recruitment and NETosis within plaques, thereby promoting plaque destabilization [60, 61]. Increased NET formation is a recognized feature of defective resolution, as NETs propagate inflammation. Our observations of elevated intracellular lipid droplets, increased cell death, and enhanced neutrophil infiltration align with dysregulated inflammatory resolution. Lipid droplets, which store polyunsaturated fatty acids, serve as substrates for both pro‐inflammatory eicosanoids and specialized pro‐resolving mediators (SPMs). Their accumulation may reflect impaired SPM biosynthesis. Although SPMs were not directly quantified here, our observations, together with prior work, suggest that the surgery‐induced APR may dampen SPM production. Such an imbalance between pro‐inflammatory and pro‐resolving lipid mediators may sustain leukocyte activation, impair efferocytosis, and disrupt plaque resolution. Collectively, these findings support a model in which surgical stress suppresses key resolution pathways, exacerbating inflammation and compromising tissue repair.

Uniquely, we observe that metabolic changes induced by postoperative inflammation cause striking and rapid changes in foam cell dynamics within atherosclerotic plaques. Due to the chronic nature of atherosclerosis, studies typically focus on long‐term changes, but our findings show that acute systemic disturbances can induce cellular changes in plaque cells as early as 24 h postoperative. This highlights the fact that the plaque microenvironment is not shielded from large systemic changes. Furthermore, this observation offers insights into the limited efficacy of clinical trials such as the AEGIS‐II CSL112 post‐MI study, where interventions were initiated up to 5 days after the physiological insult [64, 65]. In our model, lipid accumulation and foam cell apoptosis were underway at 24 and 72 h postoperatively, with significant changes in cellularity at 72 h, underscoring a critical therapeutic window that may have been missed.

Previous studies on postoperative atherosclerosis have found success in interventions that temper inflammation (e.g., statins, IL‐6 antagonism, preoperative regulatory T cell expansion) and lipid metabolism (e.g., statins) [41, 43]. However, our comparison to these studies is limited by the site of atherosclerotic plaque assessment. In those studies, plaque development in the brachiocephalic artery was minimal before surgery, and atherosclerotic plaque formation was primarily the result of the surgical procedure [41, 43]. Our results align with those of a study on postoperative atherosclerosis in the aortic root, where an increase in the necrotic core was found 15 days after orthopedic surgery [42]. Additionally, in this study, the caspase‐3 area was not different between the surgery and control groups at 5 and 15 days postoperatively, supporting our findings that apoptosis is not altered at 15 days postoperatively and that decreased apoptotic signaling likely occurs near the 5‐day postoperative timepoint [42].

Importantly, we replicate our findings of reduced cholesterol efflux to postoperative *ApoE^/−^* plasma in non‐cardiac surgery patient samples, further substantiating the translatability of our observations. Recent untargeted metabolomics of patients with and without myocardial injury after non‐cardiac surgery (MINS) identified cholesterol metabolism as a significantly altered KEGG pathway, reinforcing the critical role of lipid metabolism in postoperative cardiovascular complications [66]. Although advances in perioperative medicine have led to improved patient care and safer procedures, cardiovascular complications remain a significant concern, particularly in light of rising cardiovascular disease morbidity. A recent prospective study found that 1 in 6 patients undergoing major general surgery experienced MINS, with MINS associated with a 5‐fold increase in 30‐day mortality [67]. By understanding the acute changes in foam cell dynamics and cholesterol efflux during the postoperative period, we can better target therapeutic interventions aimed at preserving plaque stability and mitigating the risk of major adverse cardiovascular events (MACE) following surgery. With further investigation into enhancing RCT, particularly through HDL‐based therapies, we may be able to significantly reduce the incidence of postoperative cardiovascular complications, ultimately improving patient outcomes and reducing the burden of MACE in the surgical population.

## Sources of Funding

This work is supported by the Canada Graduate Scholarship‐Master's Program (D.M. Boucher and V. Rochon), the Vanier Canada Graduate Scholarship (D.M. Boucher), the UOHI Endowed Fellowship (V. Lorant, T. Laval, V. Rochon, N. Joyce), the Canadian Institutes for Health Research (PJT‐541283 and Canada Research Chair to M. Ouimet), the Heart and Stroke Foundation of Canada (M.O.), and The National Institutes of Health (R01HL171111 to S.M. Gordon).

## Disclosures

The authors have nothing to report

## Conflicts of Interest

The authors declare no conflict of interest.

## Supporting information




**Supporting File**: advs73916‐sup‐0001‐SuppMat.docx.

## Data Availability

The data that support the findings of this study are available from the corresponding author upon reasonable request.
