# Supplementary material for: Postoperative Stress Accelerates Atherosclerosis Through Inflammatory Remodeling of the HDL Proteome and Impaired Reverse Cholesterol Transport
Source: Adv Sci (Weinh). 2026 Jan 30;13(26):e11121. doi: 10.1002/advs.202511121 (PMC13159145; doi:10.1002/advs.202511121)
Supplement: Supplementary file 1 — Supporting File: advs73916‐sup‐0001‐SuppMat.docx. [file ADVS-13-e11121-s001.docx]

**

**

**Supplemental Fig.1: Gating strategy and circulating immune cells.** Circulating immune cells from blood obtained by cardiac puncture were gated for flow cytometry as follows (**a**): cells, single cells, live cells, CD45^+^ cells. Subpopulations were gated from CD45^+^ cells as follows: B cells B220^+^; cytotoxic T cells CD3e^+^CD8a^+^; helper T cells CD3e^+^CD4^+^; neutrophils CD11b^+^Ly6G^+^; inflammatory monocytes CD11b^+^Ly6G^-^Ly6C^hi^; patrolling monocytes CD11b^+^Ly6G^-^Ly6C^lo^. Cell populations as percentage of CD45^+^ cells at 24h, 72h and 15d in anesthesia control and surgery groups are represented (**b-g**). Data as min to max boxplot, # P ≤ 0.1, * P ≤ 0.05, ** P ≤ 0.01, *** P ≤ 0.001, **** P ≤ 0.0001.

**
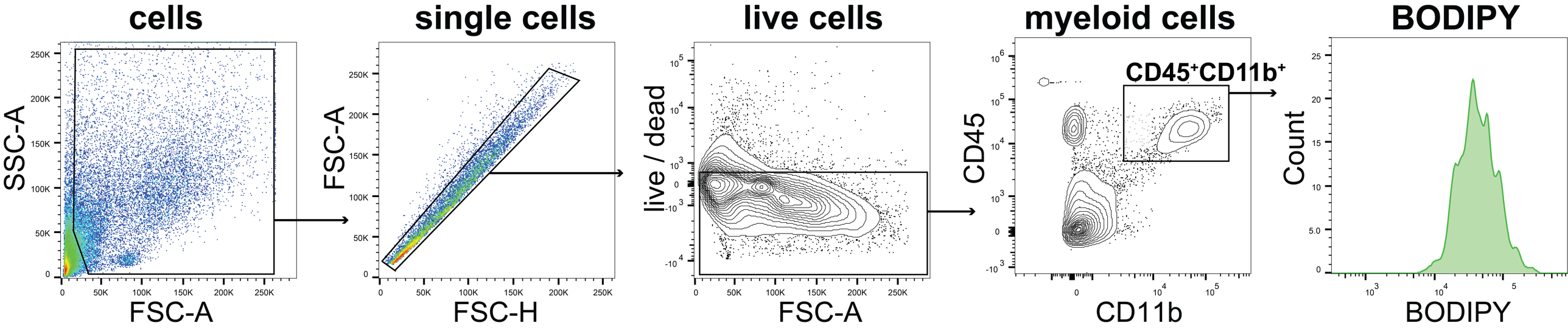
**

**Supplemental Fig.2: Gating strategy for aortic arches.** Cells from aortic digest were gated for flow cytometry as follows: cells, single cells, live cells, myeloid CD45^+^CD11b^+^ cells. Median BODIPY from CD45^+^CD11b^+^ cells was obtained to determine BODIPY MFI.

**
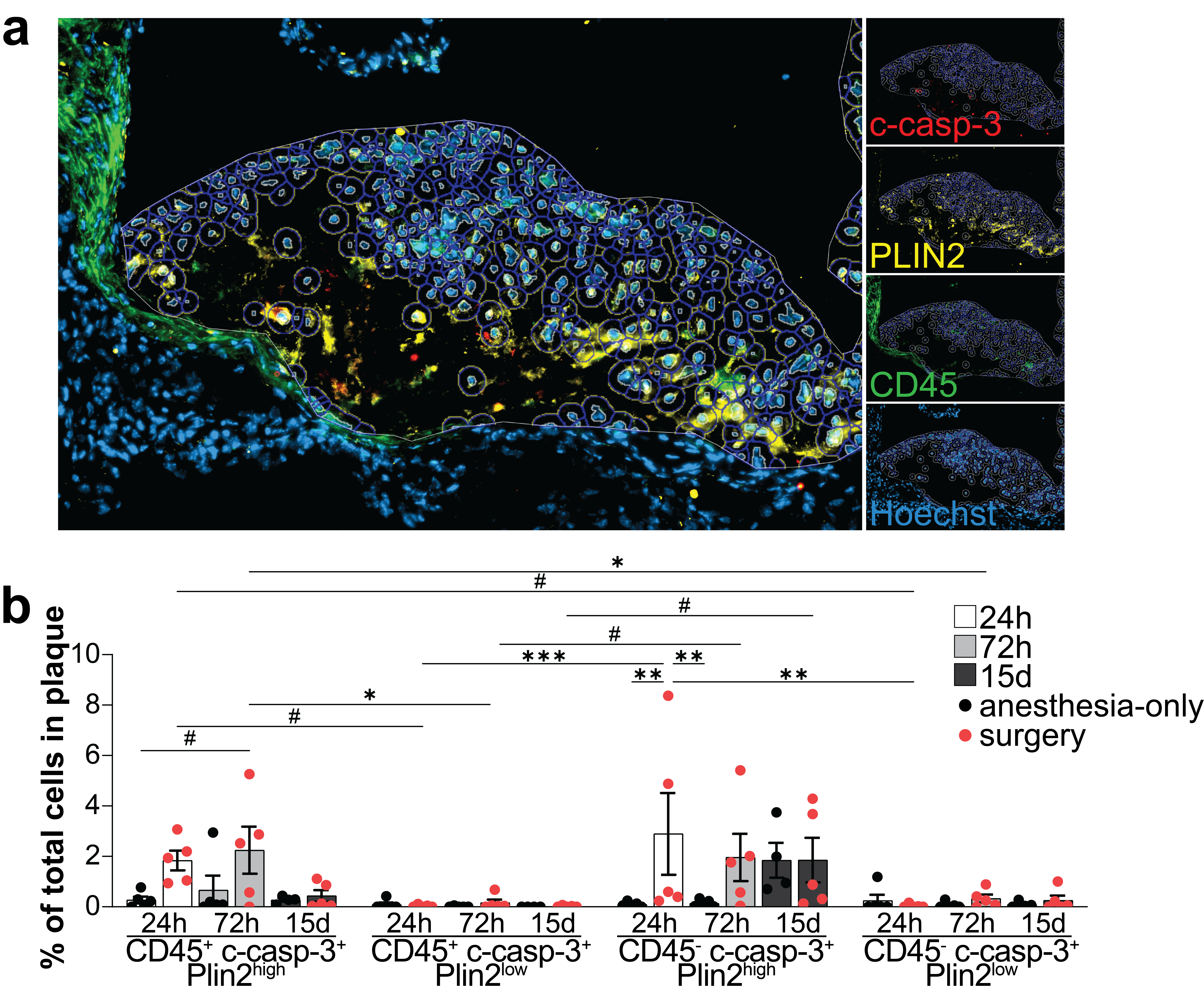
**

**Supplemental Fig.3: ZenDesk 2D image analysis cell outlines.** Fluorescence intensity of each channel was assessed by automated generation of cell outline around identified nuclei, using the ZenDesk 2D image analysis toolkit, as represented (**a**). Expanded data from **Fig.2m**. Apoptotic (c-Casp-3^+^) cell populations defined by expression of CD45 and PLIN2, expressed as percentage of total plaque cells (**b**). Data as mean ± SEM, # P ≤ 0.1, * P ≤ 0.05, ** P ≤ 0.01, *** P ≤ 0.001.


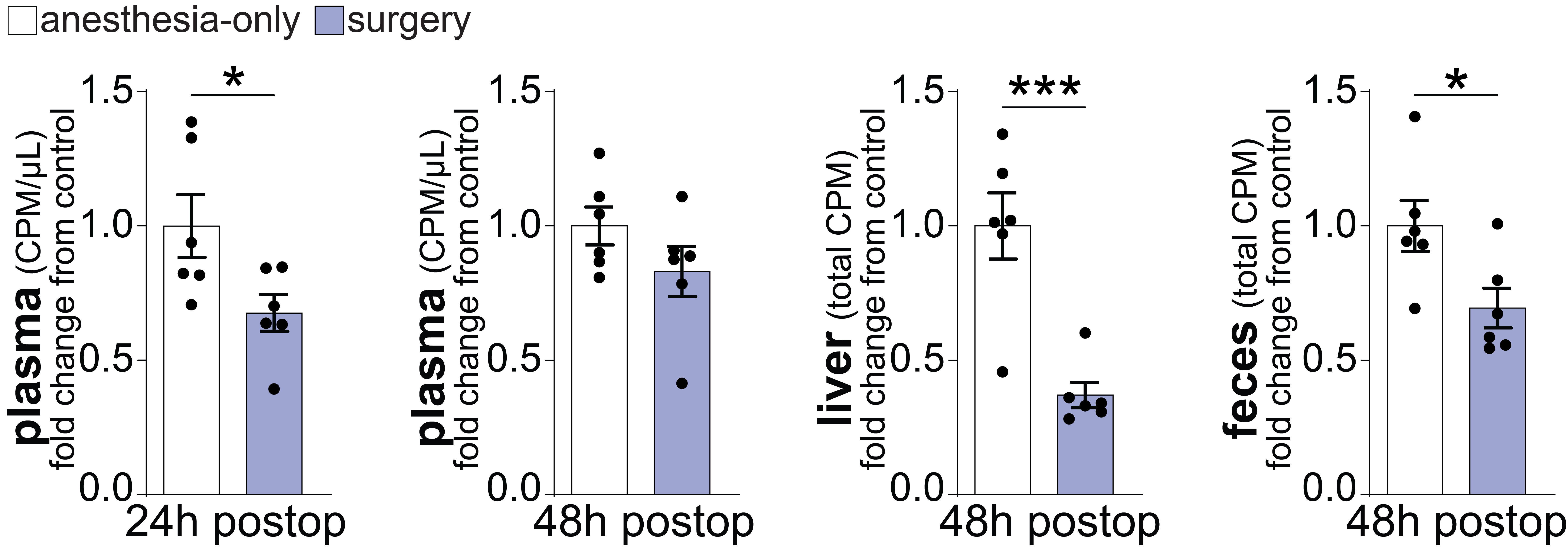


**Supplemental Fig.4: Reverse cholesterol transport in C57BL/6 mice.** 12-week-old male C57BL/6 mice (n=6/group) were randomized to anesthesia control or surgery group. BMDMs from sex- & aged-matched C57BL/6 donors were loaded with ^3^H-cholesterol-labeled agLDL for 30h and injected during recovery from anesthesia. Radioactivity was measured in plasma at 24h after surgery, and in plasma, liver & feces at 48h postoperative. Data as mean ± SEM, * P ≤ 0.05, *** P ≤ 0.001.





**Supplemental Fig.5: Hematopoietic stem and mature cell populations in the bone marrow, spleen and blood.** *ApoE*^-/-^ mice (n=5-6/group) were fed a Western diet for 8 weeks and randomized to anesthesia-only, surgery, or surgery + rApoA-I (40 mg/kg IP at surgery). Hematopoietic stem and progenitor cells (HSPCs) and mature populations were quantified in bone marrow and spleen 24h postoperatively; circulating mature cells were assessed in blood at the same time point. Populations shown: bone marrow HSPCs (**a–j**), bone marrow mature cells (**k-q**), spleen HSPCs (**r-aa**), spleen mature cells (**bb–hh**), and circulating total white blood cells (CD45^+^) (**ii**), as live cells/mL. Mature cell phenotypes are defined as in the main manuscript. Gating strategy for HSPCs is shown (**jj-qq**). HSPC subsets were defined as follows: LSK (Lin⁻CD117⁺Sca1⁺; **mm**); short-term HSC (Lin⁻CD117⁺Sca1⁺CD135⁻CD48⁻CD150⁻; **nn, qq**); long-term HSC (Lin⁻CD117⁺Sca1⁺CD135⁻CD48⁻CD150⁺; **nn, qq**); MPP2 (Lin⁻CD117⁺Sca1⁺CD135⁻CD48⁺CD150⁺; **nn, qq**); MPP3 (Lin⁻CD117⁺Sca1⁺CD135⁻CD48⁺CD150⁻; **nn, qq**); MPP4 (Lin⁻CD117⁺Sca1⁺CD135⁺CD48⁺CD150⁻; **nn, oo**); CMP (Lin⁻CD117⁺Sca1⁻CD34⁺FcγRlo; **nn, pp**); MEP (Lin⁻CD117⁺Sca1⁻CD34⁻FcγR^lo^; **mm, pp**); GMP (Lin⁻CD117⁺Sca1⁻CD34⁺FcγR^hi^; **mm, pp**); CLP (Lin⁻CD117⁺Sca1⁺CD135⁺; **mm, nn**). FMO controls used to determine gating are shown (**rr**). Data as mean ± SEM, # P ≤ 0.1, * P ≤ 0.05, ** P ≤ 0.01, *** P ≤ 0.001.

**Supplemental Table 1: List of antibodies**

| **Target** | **Conjugate** | **Clone** | **Manufacturer** | **Catalogue number** | **Tissue** |
| --- | --- | --- | --- | --- | --- |
| **Flow cytometry** | | | | | |
| CD45 | APC-Cy™7 | 30-F11 | BD Biosciences | 561037 | Blood / BM / Spleen |
| CD45R/B220 | BV786 | RA3-6B2 | BD Biosciences | 563894 | Blood / BM / Spleen |
| CD3e | APC | 145-2C11 | BD Biosciences | 553066 | Blood / BM / Spleen |
| CD4 | PerCP-Cy™5.5 | RM4-5 | BD Biosciences | 561115 | Blood / BM / Spleen |
| CD4 | PE-Cy™7 | RM4-5 | BD Biosciences | 552775 | Blood / BM / Spleen |
| CD8a | BV510 | 53-6.7 | BD Biosciences | 563068 | Blood / BM / Spleen |
| CD11b | BV421 | M1/70 | BD Biosciences | 562605 | Blood / BM / Spleen |
| Ly6G | PE-Cy™7 | 1A8 | BD Biosciences | 560601 | Blood / BM / Spleen |
| Ly6G | FITC | 1A8 | BD Biosciences | 551460 | Blood / BM / Spleen |
| Ly6C | BV605 | AL-21 | BD Biosciences | 563011 | Blood / BM / Spleen |
| CD115 | PE | AFS98 | BioLegend | 135505 | Blood / BM / Spleen |
| CD117 | BV605 | 2B8 | BD Biosciences | 563146 | BM / Spleen |
| CD34 | BV421 | RAM34 | BD Biosciences | 562608 | BM / Spleen |
| CD16/32 | PerCP-Cy™5.5 | 2.4G2 | BD Biosciences | 560540 | BM / Spleen |
| Sca-1 (Ly6A/E) | PE-Cy™7 | D7 | BD Biosciences | 558162 | BM / Spleen |
| CD135 (FLK-2) | PE-CF594 | A2F10.1 | BD Biosciences | 562537 | BM / Spleen |
| CD150 | PE | Q38-480 | BD Biosciences | 562651 | BM / Spleen |
| CD48 | APC | HM48-1 | BioLegend | 103412 | BM / Spleen |
| CD45 | APC/Fire^TM^ 750 | I3/2.3 | BioLegend | 147713 | Aortic digest |
| CD11b | BV421 | M1/70 | BD Biosciences | 562605 | Aortic digest |
| Ly6G | PE-Cy™7 | 1A8 | BD Biosciences | 560601 | Aortic digest |
| **Immunofluorescence microscopy** | | | | | |
| CD45 | Alexa Fluor® 488 Conjugate | D3F8Q | Cell signaling Technology | 59572 | Aortic sinus |
| CD45 | Alexa Fluor® 647 Conjugate | 30-F11 | Biolegend | 103123 | Aortic sinus |
| CD68 | Alexa Fluor® 750 Conjugate | FA-11 | Novus | NBP2-33337AF750 | Aortic sinus |
| PLIN2 | N/A | Polyclonal | Progen | GP42 | Aortic sinus |
| c-Casp-3 | N/A | 5A1E | Cell signaling Technology | 9664 | Aortic sinus |
| MPO | N/A | Polyclonal | R&D Systems | AF3667 | Aortic sinus |
| 3HCit | N/A | RM1001 | Abcam | ab281584 | Aortic sinus |
| Ly6G | N/A | 1A8 | BioLegend | 127601 | Aortic sinus |
| ApoA-I | N/A | Polyclonal | Invitrogen | PA5-29557 | Aortic sinus |
